# Construction and evaluation of fibrillar composite hydrogel of collagen/konjac glucomannan for potential biomedical applications

**DOI:** 10.1093/rb/rby018

**Published:** 2018-07-20

**Authors:** Jiayuan Tang, Jinlin Chen, Jing Guo, Qingrong Wei, Hongsong Fan

**Affiliations:** National Engineering Research Center for Biomaterials, Sichuan University, Chengdu 610064, P.R. China

**Keywords:** collagen, konjac glucomannan, composite hydrogel, self-assembly, fibrillogenesis, biologic evaluation

## Abstract

Konjac glucomannan (KGM) is recognized as a safe material for its health-promoting benefits and thus widely used in various fields including pharmaceutical industry. In recent decades, the combination of collagen and KGM attracts more attentions for biomedical purpose, especially the hybrid films of collagen–KGM or collagen–KGM–polysaccharide. In this study, to further and deeply develop the intrinsic values of both collagen and KGM as biomaterials, a novel kind of composite hydrogel comprising collagen and KGM at a certain ratio was fabricated under mild conditions via fibrillogenesis process of the aqueous blends of collagen and KGM that experienced deacetylation simultaneously. The chemical composition, microcosmic architectures, swelling behavior, biodegradation and dynamic mechanic properties of such resulted composite hydrogels were systematically investigated. Biologic experiments, including cell culture *in vitro* and hypodermic implantation *in vivo*, were also conducted on these collagen/KGM composite hydrogels to evaluate their biologic performances. The relevant results prove that, based on collagen self-assembly behavior, this synthesis strategy is efficient to construct a composite hydrogel of collagen/KGM with improved mechanical properties, biodegradability, excellent biocompatibility and bioactivity, which are promising for potential biomedical applications such as tissue engineering and regenerative medicine.

## Introduction

Tissue engineering involves culturing cells in biodegradable matrices and subsequently implanting into a new functional tissues [1, 2]. Scaffold materials play important roles in tissue engineering because the cell attachment, proliferation, migration and differentiation are closely effected by the biologic properties of the scaffold materials and the porous architecture of scaffolds that contribute to building appropriate microenvironments for the cell population [3, 4]. 

The materials fabricated for an ideal scaffold that functions as an analogous extracellular matrix (ECM) should be biocompatible, biodegradable and easily functionalized with bioactive components to cells [5], meanwhile, the constructed scaffold should provide a mechanical support with good interconnectivity and high porosity [1–4]. 

Collagen is the major structural protein existing in native ECM. Among various natural and synthetic macromolecules for scaffold fabrication, collagen has received extensive attentions and interest. Many researches for collagen biomaterials have proved that collagen plays an active role in mediating cellular behaviors due to its specific interactions with cells [6]. To compensate the weakness of collagen, such as poor mechanical property, the hybridization with natural biomacromolecules including polysaccharides [7–9] or synthetic polymers [10, 11] have been demonstrated to be an effective strategy to construct collagen-based hydrogel scaffolds with features similar to ECM.

Some biomaterials of collagen-based scaffolds are obtained directly by lyophilizing pure collagen or blends of collagen–polysaccharide [9, 12–14]. As this kind of scaffold preparation approach does not undergo collagen fibril reconstruction *in vitro*, these scaffolds substantially lack the structure of native fibrils, which is a crucial tension element existing in natural ECM. It is ideal that collagen–polysaccharide composites better resemble ECM in nanostructure of native-like fibrils and thus could exhibit excellent cellular activities, which was also confirmed by collagen biomaterials with the patterned features [15–17].

Fibrillogenesis of collagen *in vitro* involves the process of collagen fibril formation from its pure molecule solution under near physiological conditions, initially concentrating on the native-like fibril-containing nanocomposites for bone tissue engineering [18–20]. Actually, collagen self-assembly in the presence of polysaccharides such as alginate, chitosan and hyaluronic acid (HA) has also been well studied by the method of fibrillogenesis of these blends under mild conditions and achieved the formation of these fibrillar nanostructures [21, 22].

As an essential natural polysaccharide, konjac glucomannan (KGM) is the main component of the roots and tubers of the Amorphophallus konjac plant, which grows in mountain or hilly areas mainly in the South East of Asia [23]. KGM comprises a backbone chain of glucose and mannose monomers in a molar ratio of 1:1.6, with ∼5–10% acetyl-substituted residues at the side C-6 position [24–27]. Unlike many other biomacromolecules, the molecular weight distribution of KGM was relatively narrow and the molecular chains were extending [28]. Actually, the KGM polymer has a highly branched chain [29, 30].

Attentions for KGM has increased recently because it has been demonstrated to be effective in healthcare, such as improvement of glucose metabolism, regulation of lipid metabolism, promotion of intestinal activity and cholesterol reduction [31–33]. It is identified as a safe material according to the Food and Drug Administration (FDA) [34]. Since KGM has the characteristics of high viscosity, good water imbibing, excellent gelling and film forming, including particular biological functions, it is widely used in food, chemical, medical and pharmaceutical areas [23, 32, 35–37]. 

The findings of the benefits of KGM promote more and more attentions to be given to the applications of KGM in biomaterial field. The KGM-based hydrogel with HA was prepared from lyophilizing the mixed solution of KGM and HA to obtain the KGM/HA materials for use as a scaffold for chondrocyte cultures [38]. Stable KGM hydrogels can be formed by heating alkaline KGM solution [39, 40], which was studied and demonstrated that the deacetylation resulted by the presence of alkali is crucial for KGM gelation [41]. According to this mechanism, KGM-based hydrogel of KGM/sodium–alginate and alginate–KGM–chitosan beads were fabricated for the controlled release of drugs [42, 43]. However, it is noteworthy that almost all of the developed biomaterials containing both collagen and KGM were achieved in the form of blend films by the solvent-casting method, such as collagen–KGM or collagen–KGM–polysaccharide blend films [44–46]. A problem that cannot be ignored is that, it is difficult to maintain the bioactivity of the component of collagen due to the harsh conditions of high temperature or high temperature along with the existence of alkali in the film preparation process.

Collagen macromolecules are characteristic of rigidity. Accordingly, the hydrogel formed by collagen molecule self-assembly is a typical rigid hydrogel. On the other hand, KGM is a type of nonionic polysaccharide with abundant hydroxyls. Thus KGM and collagen are miscible in a wide range of pH values. Furthermore, the main chains of KGM polymer are highly branched and semi-flexible [28]. After the treatment of deacetylation, some changes will occur to the molecular structures and result in more flexible molecular main chains of KGM [39–41, 47]. Based on these individual features of collagen and KGM, we designed to achieve a novel composite hydrogel by introducing KGM macromolecules into collagen fibrillar matrix. This synthesis process utilizes the characteristic fibrillogenesis behavior of collagen macromolecules rather than the gelling process of KGM involving high temperature with the existence of alkali. Therefore, this composite hydrogel of collagen/KGM can be constructed under mild conditions. What is more significant is that the interweaving of flexible and branched KGM macromolecules into three dimensional (3D) network of collagen fibrils produce a good physical crosslinking effect, which endows as-obtained collagen/KGM composite hydrogel with obviously improved mechanical properties, which is the main important objective of the present work. Developing such a novel composite hydrogel of collagen/KGM via the approach of collagen fibrillogenesis under mild conditions is also promising because the natural bioactivities of collagen molecules are preserved.

Although collagen and KGM have received extensive focus and explorations for various biomedical purpose [38, 46, 48, 49], including hybrid films derived from the solution blends of collagen and KGM, the preparation route of combining collagen with KGM in the form of hydrogel from the angle of collagen self-assembly feature to obtain a composite hydrogel with collagen-based hybrid fibrillar networks has rarely been reported so far. This facile synthesis strategy can further broaden and deepen the exploration of the composites of collagen/KGM without just being limited to the form of their hybrid films in biomedical field. Such developed composite hydrogel is a platform biomaterial which has various potential uses as supporting scaffolds, surgical repairing, wound dressing and carrier materials for tissue engineering, medical regeneration and drug delivery.

## Materials and methods

### Materials and reagents

Acid soluble collagen (Type I) with high purity was extracted from calf skin according to the procedures described by Miller, with some improvements of our laboratory [50]. KGM powder (purity ≥90%, 200-mesh, Sheli Ltd, Hongkong, China) was further purified after received to obtain the final purity of no <98% in all of our experiments. Fluorescein diacetate (FDA), propidium iodide (PI) and collagenase (Type I) were purchased from Sigma-Aldrich (St Lous, MO, USA). Hydroxyproline assay kit was obtained from Nanjin Jiancheng Co., Ltd, China. Cell Counting Kit-8 (CCK-8) was provided by KeyGEN BioTECH (Nanjing, China). Human 6-ketoPGF1a enzyme-linked immunosorbent assay (ELISA) kit and Human TXA2 ELISA kit were provided by Fushun Industrial Co., Ltd (Shanghai, China). RPMI Medium (Hyclone), fetal bovine serum (FBS, Gibco), trypsin (Hyclone), penicillin–streptomycin solution (Hyclone) and phosphate buffer solution (PBS, Hyclone) were all used in cell culture experiments. Pentobarbital sodium (Sigma) was used in animal experiment. Ammonia water (30%, w%), polyethyleneglycol (PEG, Mw 20 000), acetic acid and other chemicals (Kermel Ltd, Chengdu, China) were analytical grade reagents and used as received. The ultrapure water from a Milli-Q system was used in all procedures of our experiments.

### Construction of collagen/KGM composite hydrogels

An aqueous KGM solution with the concentration of 0.6% (w%) was added dropwise to an acetic acid collagen solution (3.0 mg ml^−1^, pH 3.5) with medium strong stirring in ice bath. The ratio of collagen to KGM was 6:4 (w/w) in the composite system. After dropping KGM solution, stirring was continued for ∼20 min. The mixture suspension was then degassed by means of centrifugation and transferred into a dialysis bag (Mw cutoff: 3500), which was placed into an aqueous PEG solution to concentrate the composite solution of collagen/KGM, with subsequent additional PEG being added again to expedite the concentrating process until the concentration of collagen arrived ∼5.6 mg ml^−1^. Then, the concentrated composite solution was injected into a certain module such as beaker, which was placed in an enclosed vessel filled with ambience of ammonia supplied by a vial of ammonia water inside the vessel. When the pH of the composite solution of collagen/KGM was increased to 8.5–9.0, the module was taken out of the vessel and incubated in a water bath at 37°C for 4 h. All experimental procedures involving collagen were conducted at 4°C.

Fibrillogenesis of the mixture system was initiated by raising temperature, resulting in the gradual assembly of a composite hydrogel of collagen/KGM. As-obtained hybrid hydrogels were thoroughly rinsed in super-purified water to remove residual salts for subsequent experiments or analyses.

### Characterization

#### Fourier transformation infrared spectroscopy

To perform precise investigations for chemical components and structures of the composite hydrogels, a NEXUS 670 instrument (Thermo Electron, USA) was employed for Fourier transform infrared spectroscopy (FTIR) analyses, running in the range of 4000–400 cm^−1^ with a resolution of 1.0 cm^−1^ and 20 scan accumulations.

### Scanning electron microscopy

The microstructure of the composite hydrogel of collagen/KGM was analyzed using a field-emission scanning electron microscope (FE-SEM, S-4800 Hitachi). The specimens for SEM were applied to conductive adhesive directly with Au sputter-coating.

### Dynamic mechanical measurement

The thermomechanical properties including storage modulus and loss modulus were tested on a dynamic thermomechanical analysis machine (TA Instruments Q600, USA) at 37°C. Every hydrogel sample received a stress with a set of test frequency of 1, 2 and 5 Hz and vibration amplitude of 20 mm during the test process. Data scan was repeated five times at every frequency. The size of all hydrogel samples were modeled in 8 mm diameter with 3 mm thickness.

### Circular dichroism spectra analyses

To identify that the natural structures of the collagen macromolecules in the collagen/KGM composite hydrogel were still well maintained or not, circular dichroism (CD) spectra (Chirascan plus spectrometer, CSP20096, Great Britain) were employed to perform the analyses. The hydrogels were dissolved in acetic acid (pH 2.5), and the solution was diluted to attain the collagen concentration of 0.25 mg ml^−1^. The measuring parameters were scanning range of 190–260 nm, spectral width of 1 nm, resolution of 0.1 nm, scanning speed of 100 nm min^−1^ and repetition of four times.

### Swelling behavior investigation

The tested samples were sponge scaffold discs (diameter: 7 mm, thickness: 2 mm) obtained from the lyophilized hydrogels. These samples were weighed as Wdry (Wd), and then immersed in 10 ml PBS (pH 7.4) at 37°C for a period of time. At the soaking intervals of 1, 3, 6, 9, 12, 24, 36 and 72 h, the swollen samples were removed from the PBS, blotted with a filter paper to absorb the excess liquid, and weighted as Wswelling (Ws). The swelling ratio (S) of each sample at every soaking interval was calculated according to this formula:

$$S=\left[\frac{\text{Ws}-\text{Wd}}{\text{Wd}}\right]\times 100\%.$$

### Enzymatic degradation

*In vitro* biodegradation tests of the hydrogels were performed by using collagenase (I) digestion, in which the hydroxyproline analyses were used to quantitatively detect the amount of collagen component degraded from the tested samples. The measuring process was according to the procedures described in the instruction of the hydroxyproline assay kit. Briefly, each group of the hydrogel scaffold samples was soaked in PBS (pH 7.4, 0.01% azide) containing 0.2 mg ml^−1^ collagenase at 37°C.The enzymolysis was stopped at a given time via cryopreservation of the assay mixture at −25°C immediately for ∼1 h and then incubating at 2°C. After centrifugation at 1500 rpm for 10 min, 1 ml supernatant was pipette and transferred into a glass stopper equipped test tube containing 6 M HCl and hydrolyzed at 110°C for 18 h. The hydroxyproline released from the hydrogel scaffold was detected at the absorbance of 550 nm on a spectrometer (Lambda650, PE Ltd, USA) by using a standard curve.

### Cell culture

Studies of cytocompatibility and cellular behaviors were performed on the composite hydrogel of collagen/KGM. All the hydrogel samples (thickness: 3 mm) were directly prepared in the wells of 24-well culture plates under aseptic conditions. Human umbilical vein endothelial cells (HUVECs) were chosen for this evaluation experiments. Before cell seeding, the material samples were rinsed thoroughly with sterile PBS, then immersed in RPMI medium overnight in the 37°C incubator. HUVECs of the third passage were harvested by trypsinization using a 0.05% trypsin/EDTA, counted and resuspended in complete medium; 1 × 10^5^ cells in 1 ml culture medium were seeded onto the pretreated hydrogel samples. Then, the cell-inoculated hydrogels were cultured statically in an incubator with changing the medium every 2 days.

### Proliferation and attachment HUVECs

The viability of HUVECs seeded on the hydrogels was examined by using CCK-8. HUVECs were seeded on the surface of collagen/KGM composite hydrogel as the sample group, on the surface of pure collagen hydrogel as the control group and well culture plate as the blank control group, respectively, with 1 × 10^5^ cell density. When they were cultured to 1, 3 and 7 days, 900 µl serum-free medium and 100 μl CCK-8 reagent were added. After continuous incubation for another 3 h away from light, 100 μl supernatant was absorbed from all samples of each group, and transferred to 96-well plate (pay attention to avoid bubbles), then the absorbance of these orange-red supernatants was recorded at 460 nm on a microplate reader (Bio-Rad 550 spectrometer).

The HUVEC morphology and spreading onto the hydrogels after 1, 3 and 7 days of culturing time were examined by FE-SEM observations. The cell-cultured hydrogels were washed with PBS and then fixed in 2.5% glutaraldehyde for 48 h at 4°C, then soaked again in PBS for 10 min with repeating five times to remove the residual glutaraldehyde. Subsequently, the fixed samples were dehydrated with a series of graded ethanol, and then soaked in isoamyl acetate two times with each time of 10 min to replace the residual ethanol in the samples. After treated by CO_2_ critical point drying, these dried samples were sputter-coated with Au for SEM observations.

### Growth of HUVECs on the hydrogels by confocal microscopy and bioactivity detection by ELISA

Fluorescent staining of FDA/PI was performed for HUVECs growing on the surface of the hydrogel materials to investigate their survival state. HUVECs were inoculated (density 1 × 10^5^) on collagen gel surface and composite gel surface, respectively. When cultured to 1, 3 and 7 days, the cell-cultured hydrogels were washed with PBS and then soaked in FDA/PI (25 μg ml^−1^ FDA and 25 μg ml^−1^ PI) staining solution for 5 min, then washed with PBS to remove the uncombined FDA/PI. Fluorescence of stained HUVECs was achieved on a Leica SP5 confocal laser scanning microscope (CLSM) at the excitation/emission wavelength of 490/520 nm for FDA and 540/625 nm for PI.

ELISA kits were used for quantitative determination for the concentrations of cytokines PGI2 and TXA2 released during the growth process of HUVECs on the surface of the hydrogels. When HUVECs were cultured to 1, 3 and 7 days, 10 μl cell culture supernatant was taken and added with the diluent in accordance with the kit instructions. Through a series of repeated operations including incubation, rinsing, reaction with the enzyme reagent and developing, the optical density (OD) value of the supernatants was measured at 450 nm wavelength. Then the OD values were substituted into the regression equation of the standard curve to calculate the concentrations of cytokines PGI2 and TXA2 secreted in the culture solutions corresponding to different culture time.

### Subcutaneous implantation for biological evaluation *in vivo*

In this work, all animal studies were carried out in compliance with the guidelines formulated by the Institutional Animal Care and Use Committee (IACUC). Six-week-old, healthy male SD rats were adopted to conduct subcutaneous implantation of our hydrogel materials to investigate the biocompatibility and the potential tissue repairability of these hydrogels *in vivo.*

Pentobarbital sodium solution (3%) was intraperitoneally injected into the rats for anesthesia (30 mg kg^−1^). After being shaved, the back skins were thoroughly sterilzied using iodophor and 75% ethanol. Then incisions were made on the rat backs, and the samples (diameter: 10 mm thickness: 3 mm) of the composite hydrogel and the pure collagen hydrogel were subcutaneously impltanted through the incisions, respectively, then wounds were sutured. The postoperative state of food intake and wound healing of the experimental animals were observed and recorded. On the postoperative seventh day, the implanted materials along with the dermal tissues around the implants were harvested, washed in PBS, fixed in 4% paraformaldehyde solution for 24 h and dehydrated with a graded series of ethanol solutions. After performing paraffin embedding and sectioning for these dehydrated tissues, the obtained tissue sections were stained using hematoxylin–eosin (HE) for microscopic examinations and analyses.

### Statistical analysis

Statistically significant differences between groups were detected by one-way analysis of variance at a confidence interval of 95%.

## Results and discussion

### Functional groups analyses by FTIR

**Figure 1.**
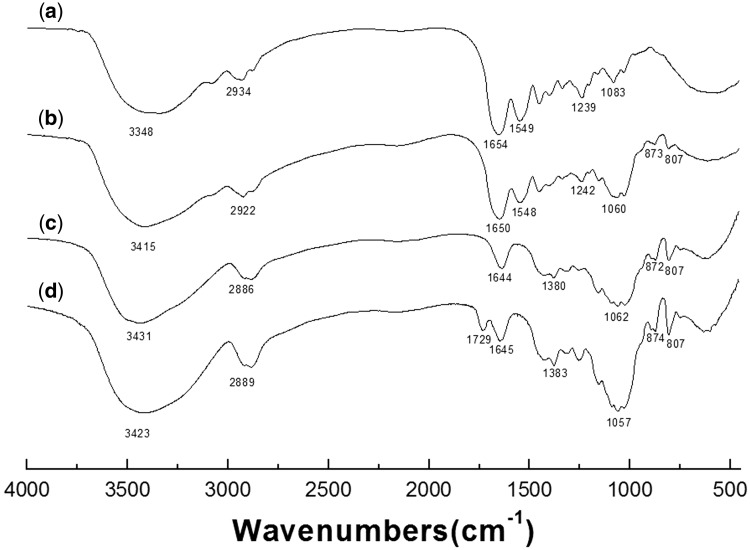
FTIR Spectra of (**a**) pure collagen, (**b**) collagen/KGM composites, (**c**) d-KGM and (**d**) KGM

As shown in Fig. 1, the FTIR spectra show the typical signals of polysaccharide along with the signals of collagen. The absorption bands at 3391–3430 cm^−1^ were from the stretching vibrations of the abundant hydroxyls of the polysaccharide of KGM (Fig. 1d) [51]. The bands at 873, 872 and 874 cm^−1^ were derived from the characteristic vibrations of β-d-glycosidic bonds in collagen/KGM composite, deacetylate- KGM (d-KGM) and KGM, respectively (Fig. 1b–d) [47]. And the band at 1730 cm^−1^ attributed to the presence of acetyl groups was identified in KGM (Fig. 1d) but not in both d-KGM (Fig. 1c) and collagen/KGM composite gels (Fig. 1b) [47, 51], which suggests the complete removal of acetyl groups from the component of KGM under alkaline condition during the preparation process of the collagen/KGM composite hydrogel. The absorption peaks at 807 cm^−1^ were attributed to the breathing vibrations of pyran rings within KGM molecule chains [47], and the bimodal bands around 1057 cm^−1^ were derived from the characteristic vibrations of primary hydroxyl groups on the pyran rings of native KGM macromolecules (Fig. 1d). The same bimodal bands around 1060 and 1062 cm^−1^ for collagen/KGM composite and d-KGM, respectively, take a mild red shift (Fig. 1b and c), implying some variations occurred for the hydrogen bonds. In accordance with the typical amide vibrational bands (1654, 1549 and 1239 cm^−1^ for amide I, amide II and amide III bands, respectively) [52, 53] detected in the pure collagen (Fig. 1a), the band complexes of amide were also obviously identified in the composite hydrogels of collagen/KGM, with the peaks at 1650, 1548 and 1242 cm^−1^ for amide I, amide II and amide III, respectively (Fig. 1b), and also with some red shift. Main characteristic absorption bands of collagen and KGM all can be found in the FTIR analysis of the collagen/KGM composite, except for that of acetyl groups of KGM. These results give the information that after the mixture of collagen and KGM experienced the process of fibrillogenesis, the stretching vibrations of hydroxyls became enhanced and took red shift, indicating the enhancement of the hydrogen bond interactions occurred between these two macromolecules. Accordingly, the hydrogen bonds play an essential role in the compatibilization of the collagen-KGM blend system.

### Morphologies and microstructures of the hydrogels

**Figure 2.**
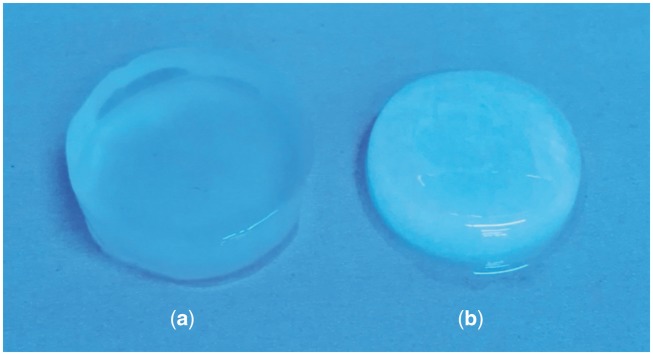
Images of macroscale morphology recorded by a camera showing (**a**) collagen/KGM composite hydrogel and (**b**) pure collagen hydrogel

**Figure 3.**
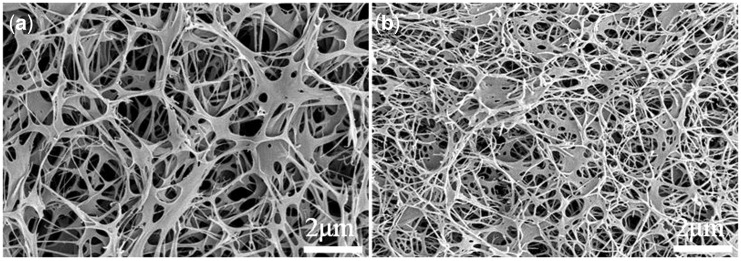
SEM Images showing the microstructure morphology of (**a**) collagen/KGM composite hydrogel scaffold and (**b**) pure collagen hydrogel scaffold

Figure 2 shows the macroscopic views of a collagen/KGM composite hydrogel and a pure collagen hydrogel. The composite hydrogel constructed by thermosensitive fibrillogenesis of a collagen–KGM blend system in which collagen is the dominant component has a transparent appearance with good elasticity (Fig. 2a), while the pure collagen hydrogel present an opaque appearance and also has a certain degree of elasticity (Fig. 2b). The explanation for this obvious variance in their macroscopic appearance can be found in the comparison of the microstructures shown in SEM images (Fig. 3). Morphological features given in Fig. 3a reveal that, resembling the pure collagen hydrogel, the composite hydrogel of collagen/KGM presents an evenly distributed and porous structure, suggesting good macromolecular compatibility between collagen and KGM, and that so-obtained composite fibrils exhibit the same good connectivity and integration as the pure collagen fibrils. The fibrils of the composite hydrogel are generally wider in diameter than the fibrils of the pure collagen hydrogel. According to the measured data of the mean fibril size, this value for the pure collagen hydrogel was 179 ± 21 nm, while the mean fibril size of composite hydrogel was 251 ± 32 nm. But the diameter size of the fibrils of the pure collagen hydrogel is comparatively more uniform than the diameter size of the fibrils of the composite hydrogel. This result indicates that a high proportion of wide fibrils were generated in the composite hydrogel compared with the pure collagen hydrogel. Both kinds of hydrogel all exhibit a porous microstructure of 3D networks with pore size range of 200–2600 nm and 130–2240 nm for collagen/KGM composite hydrogel and pure collagen hydrogel, respectively. Accordingly, such a fibrillar network of the composite hydrogel appears to possess more excellent elasticity than the relatively fine and closely woven network microstructure of the pure collagen hydrogel.

### Dynamic mechanical behaviors

**Figure 4.**
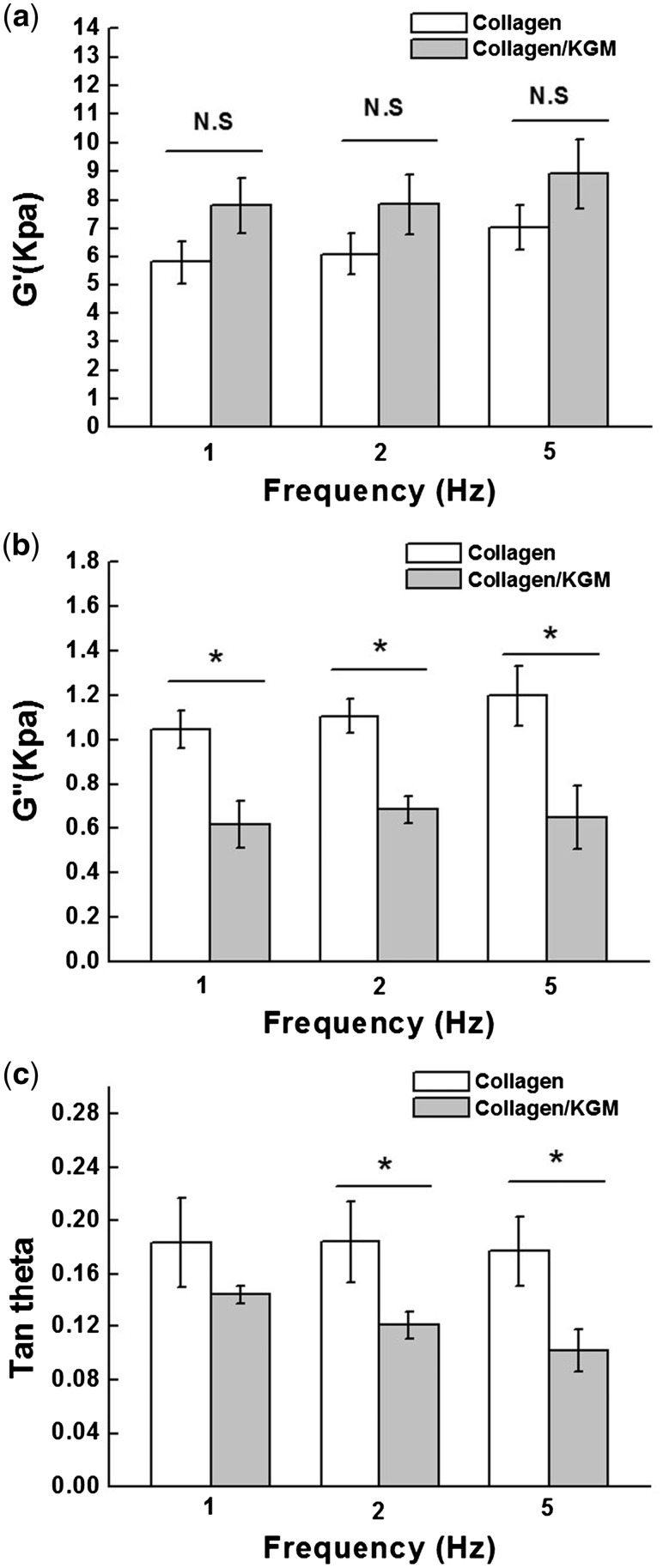
The dynamic mechanical property tests of collagen/KGM composite hydrogels and pure collagen hydrogels. The dependence of (**a**) storage modulus (*G*′), (**b**) loss modulus (*G*″) and (**c**) average values of tan (*θ*) on the testing frequency. Asterisk (*) indicates *P* < 0.05, N.S., not significant; *n* = 3 per group

Storage modulus and loss modulus are the main parameters to investigate the elasticity and viscosity of hydrogels. Tan (*θ*), which is used to characterize the nature of toughness, is a ratio of loss modulus (*G*″) value to storage modulus (*G*′) value. The measurement results (Fig. 4) show that compared with the pure collagen hydrogels, the collagen/KGM composite hydrogels exhibit enhanced elasticity and statistically significant decrease of viscosity at each testing frequency, suggesting that by combining collagen with KGM, as-obtained collagen-based composite hydrogel of collagen/KGM was evidently improved in terms of its mechanical properties. The explanation for this result could be the good physical crosslinking effect originated from the interweaving of flexible and branched KGM macromolecules into collagen fibrillar network. This improvement of the dynamic mechanical properties of hydrogels also can be found in the microstructure difference between the pure collagen hydrogel and the composite hydrogel of collagen/KGM, which has relatively larger sizes of fibrils and pores (Fig. 3a). These results reflect the principle that the performance of materials is significantly determined by their structures.

### CD analyses

**Figure 5.**
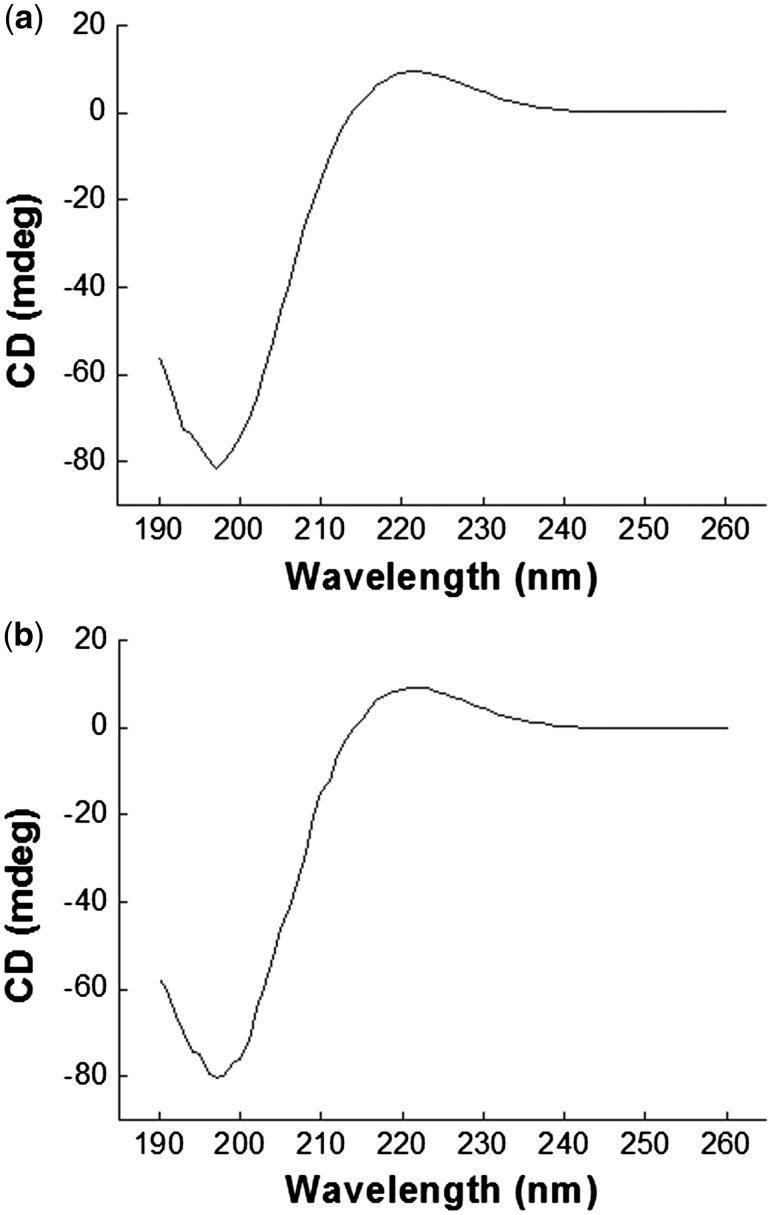
CD analysis spectra of (**a**) the collagen/KGM composite hydrogel and (**b**) the pure collagen hydrogel

CD spectroscopy is an effective approach for characterization of the secondary structures of proteins. Spectra within far-ultraviolet region usually reflect the structural state of peptide bonds, which have a highly regular arrangement to form a particular secondary structure of protein. A certain secondary structure determines and corresponds to its specific CD spectra. The CD spectra measured from collagen/KGM composite hydrogel and pure collagen hydrogel are shown in Fig. 5a and b. An intense positive peak at wavelength of ∼220 nm and an intense negative peak at ∼197 nm appeared for both the composite hydrogel and the pure collagen hydrogel as standard control. These two spectrums almost highly coincide and exhibit the characteristic CD spectrum of the secondary structures of triple helix of collagen macromolecules. Accordingly, complemented by IR analyses (Fig. 1), it can be confirmed that the natural configurations of triple helix of the collagen macromolecules in the collagen/KGM composite hydrogel were well maintained during the synthesis process under mild conditions in this works. This means that the composite hydrogels of collagen/KGM possess bioactivity originating from the component of collagen.

### Swelling behavior

**Figure 6.**
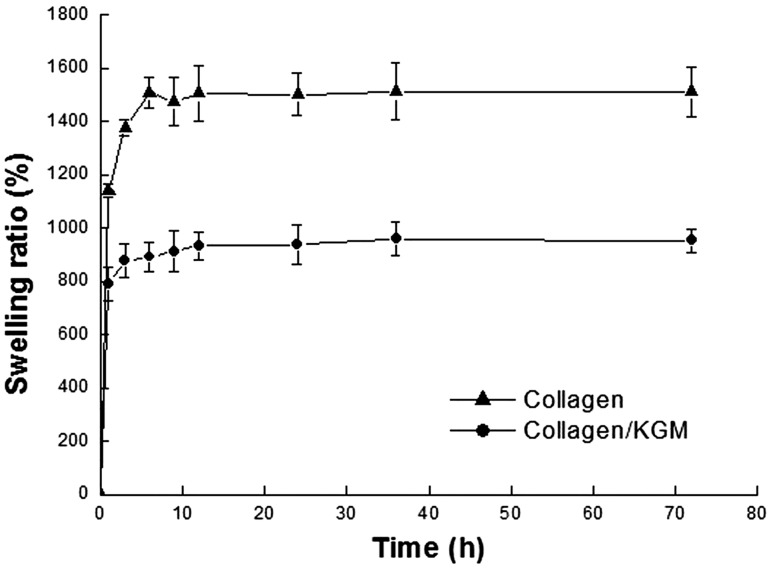
Swelling kinetics of the collagen/KGM composite scaffolds and the pure collagen scaffolds

The samples used to determine the swelling behaviors of the lyophilized hydrogels were sponges that were derived from the composite hydrogel of collagen/KGM and the pure collagen hydrogel by lyophilization. Figure 6 is the swelling kinetics of theses sponges in PBS at 37°C. During the early incubation of 6 h, a rapid swelling occurred. After 12 h, the swelling tendency attenuated for both samples. The equilibrium swelling ratio at 72 h was 9.3 and 14.6 for the composite hydrogel-derived sponge and the pure collagen hydrogen-derived sponge, respectively. The pure collagen hydrogen-derived sponge has a good water absorption capability reaching about 15 times of its own weight due to the existence of rich hydrophilic groups such as amidogen, carboxyl and hydroxyl on collagen molecular chains. KGM is also a hydrophilic polysaccharide with plentiful hydroxyl groups [23, 37]. Accordingly, it is reasonable that the collagen/KGM composite hydrogel-derived sponge scaffold containing KGM should have an enhanced water-uptake capacity. However, compared with the pure collagen hydrogel-derived sponge scaffold, the scaffold derived from as-obtained collagen-based composite hydrogel exhibits a greatly reduced water absorption capability. This obvious decrease of the swelling ratio was ascribed to the formation of abundant hydrogen bonds between the carboxyl/amidogen groups on collagen molecules and the hydroxyls on KGM molecules after the molecule combination of KGM and collagen. Consequently, the hydrophilic groups which can absorb free water molecules by forming hydrogen bonds were partly occupied, resulting in reduced swelling ratio of the collagen/KGM composite hydrogel-derived sponge scaffold.

### Enzymatic degradation behaviors

**Figure 7.**
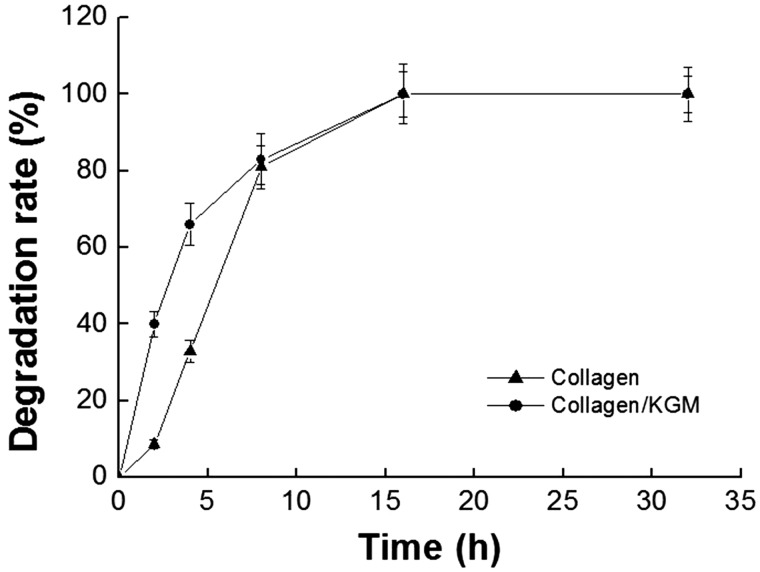
Enzymatic degradation of the collagen/KGM composite hydrogels and the pure collagen hydrogels in PBS containing collagenase of 0.2 mg ml^−1^ at 37°C

The biodegradation data of the collagen/KGM composite hydrogel and the pure collagen hydrogel are presented in Fig. 7. In hydrogel state, the composite scaffold of collagen/KGM degraded more quickly than the pure collagen scaffold in initial 6 h. After incubation for ∼16 h, complete degradation almost reached for both hydrogels. Compared with the fibrillogenesis of pure collagen macromolecule system, there were a number of KGM macromolecules distributing among the collagen molecules in the collagen/KGM composites, the whole or a part of the KGM macromolecular chains were combined in the obtained collagen fibrils during the fibrillogenesis progress of the system of collagen-KGM, which is the reason for the morphology difference between collagen/KGM composite fibrils and pure collagen fibrils (Fig. 3). And the short range order of the composite fibrils of collagen/KGM is not better than the short range order of pure collagen fibrils, which results in an exposure of more collagenase recognizable sites of the collagen molecules in the solution. Consequently, the degradation of the collagen/KGM hydrogel proceeded at a faster rate in the initial degradation stage. After 8 h of incubation, both composite hydrogel and pure collagen hydrogel were broken into small blocks, in which there was no obvious difference for the number of collagenase recognizable sites on the collagen molecules, resulting in almost the same degradation rate after 8 h of incubation.

### Cell growth, proliferation and morphology on the surface of hydrogel materials

**Figure 8.**
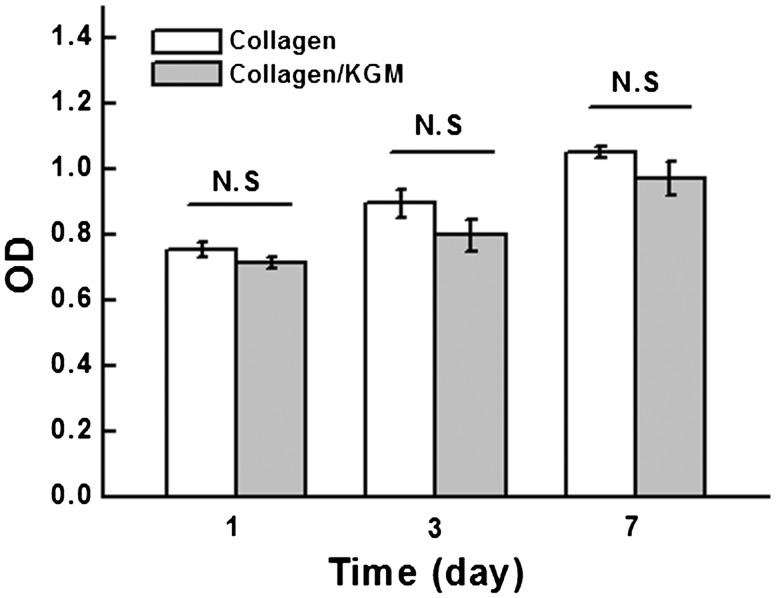
Measurements by cell counting kit-8 for HUVECs proliferation on the surface of collagen/KGM composite hydrogel and pure collagen hydrogel. N.S., not significant; *n* = 3 per group

Under the existence of electron-coupling reagent, WST-8 contained in CCK-8 reagent could be reduced by dehydrogenase to produce highly water-soluble orange-yellow formazan products, the quantity of which was directly proportional to the cellular proliferation. Its light absorbance determined at 450 nm wavelength on ELISA detector could indirectly reflect the quantity of viable cells. The results in Fig. 8 show that the group of composite hydrogel of collagen/KGM shows an approximate cellular proliferation rate to the group of pure collagen hydrogel, which presented obvious ascending tendency with the culture time. The cellular proliferation rate tended to be stable at 3 day and reached a peak value at 7 day, indicating that compared with the pure collagen hydrogel which is recognized as a kind of biomaterials with excellent biocompatibility, the composite hydrogel of collagen/KGM also has good cytocompatibility with promoting cell proliferation.

**Figure 9.**
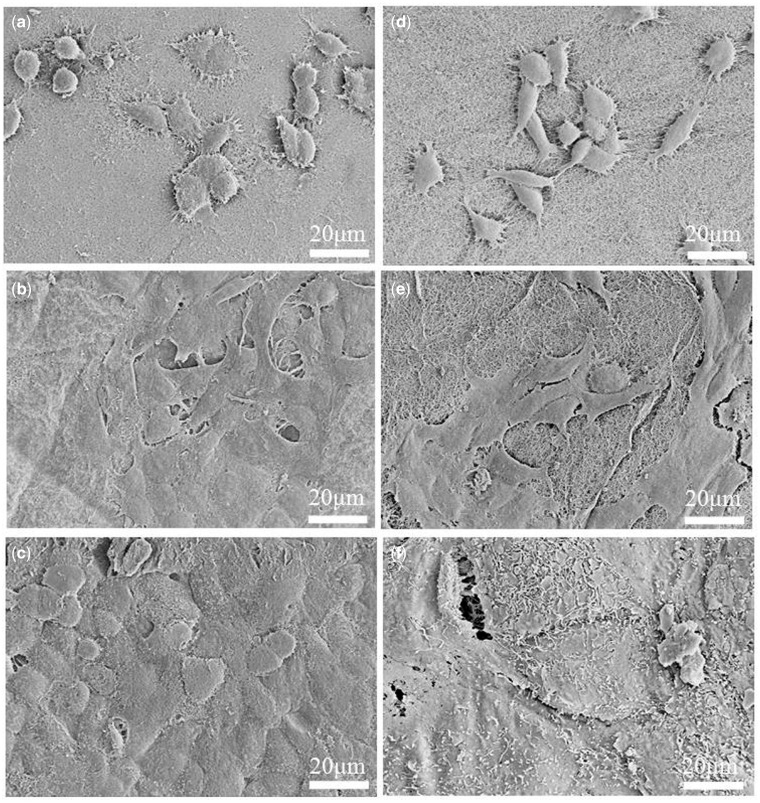
FE-SEM Micrographs representing cell attachment and growth after culturing for 1 day (**a** and **d**), 3 days (**b** and **e**) and 7 days (**c** and **f**) on the surface of the collagen/KGM composite hydrogels (a–c) and the pure collagen hydrogels (d–f)

As shown in SEM micrographs (Fig. 9), the HUVECs cultured on the composite hydrogel of collagen/KGM for 1 day had closely attached on the material surface (Fig. 9a and d). Cells present irregular ellipsoidal or fusiform shape and stretched out a lot of pseudopodia, and the cell density was relatively low. After 3 days, cells were tiled and spread on the material surface, the contact between cells was very close and the cell density obviously increased (Fig. 9b and e). As the culture prolonged to 7 days, the cells experienced high-degree differentiation with great density and were completely tiled and spread (Fig. 9c and f). These HUVECs were interconnected to form flaky shape and closely adhered to the hydrogel material surface and became a whole with the material. This morphological variation of the cell growth indicates that HUVECs could compatibly grow on the surface of the composite hydrogel of collagen/KGM, which possesses an excellent cytocompatibility of promoting cell adhesion, spreading and differentiation. These results hint that the composite hydrogel of collagen/KGM in this study maintains all the bioactivities owned by pure collagen hydrogel itself, even improved somewhat.

**Figure 10.**
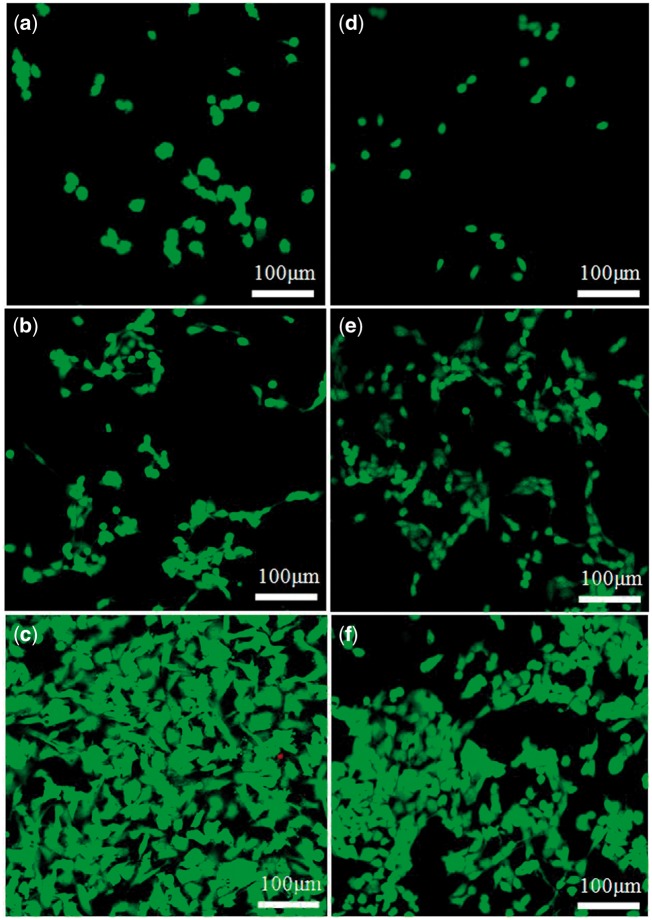
Confocal micrographs stained with FDA/PI showing HUVECs growth and attachment after culturing for 1 day (**a** and **d**), 3 days (**b** and **e**) and 7 days (**c** and **f**) on the surface of collagen/KGM composite hydrogels (a–c) and the pure collagen hydrogels (d–f)

The information conveyed from the images of CLSM (Fig. 10) is consistent with the results of CCK-8 determination, namely the growth and proliferation of the HUVECs on the surfaces of the composite gel of collagen/KGM and the pure collagen gel present an ascending tendency with the culture time, and cells had grown to contact each other when cultured for 7 days (Fig. 10c and f). There are very few dying cells on both hydrogels after rinsing. On the first culture day, the quantity of the cells on both material surfaces was small and most presented spherical distribution. When cultured for 7 days, the cell spreading and differentiation status was obvious and normal, and the cell number increased greatly, which is completely consistent with what observed under SEM. It is verified again that the collagen/KGM composite hydrogel constructed in this work could support the adhesion and growth of HUVECs with superior biocompatibility.

### The functional effect of the composite hydrogel on HUVECs

Prostacyclin PGI2, an antagonist of thromboxane TXA2, has the anti-platelet aggregation effect and vasodilatation effect. If synthesis of PGI2 decreases, it will increase the possibility of thrombosis. The experimental measurement results in Table 1 show that PGI2 could be detected since the first culture day in all groups of composite hydrogel, pure collagen hydrogel and blank control of 48-well plate. With the extension of the culture time, PGI2 secretions in the culture solutions of these groups all present a descending tendency. After 7 days of culture time, PGI2 concentration in the blank control group decreased to the most extent, while the value of PGI2 concentration in the composite hydrogel group was the highest among the three groups. Anti-PGI2 thromboxane TXA2 was not detected in all culture solutions of all experiment groups due to its excessively low content. These data suggest that the composite hydrogel of collagen/KGM possesses cell affinity and also favorable supporting behavior for the normal functional expression of HUVECs. Moreover, it seems that the biological properties of the composite hydrogel show a tendency of being superior to that of the pure collagen hydrogel to a certain degree, because high value of PGI2 means good potential of preventing the occurrence of thrombus.

**Table 1.** Concentration of prostacyclin detected in the culture solutions (pg ml^−1^)

PGI2	1 day	3 days	5 days	7 days
collagen/KGM	76.36 ± 3.36	67.94 ± 2.80	62.26 ± 1.95	61.10 ± 5.30
collagen	67.94 ± 6.68	64.77 ± 7.00	63.37 ± 7.96	56.80 ± 11.48
blank control	74.08 ± 9.00	66.41 ± 6.96	55.71 ± 4.28	53.01 ± 7.12

(*n* = 3).

### Biological assessment *in vivo*

**Figure 11.**
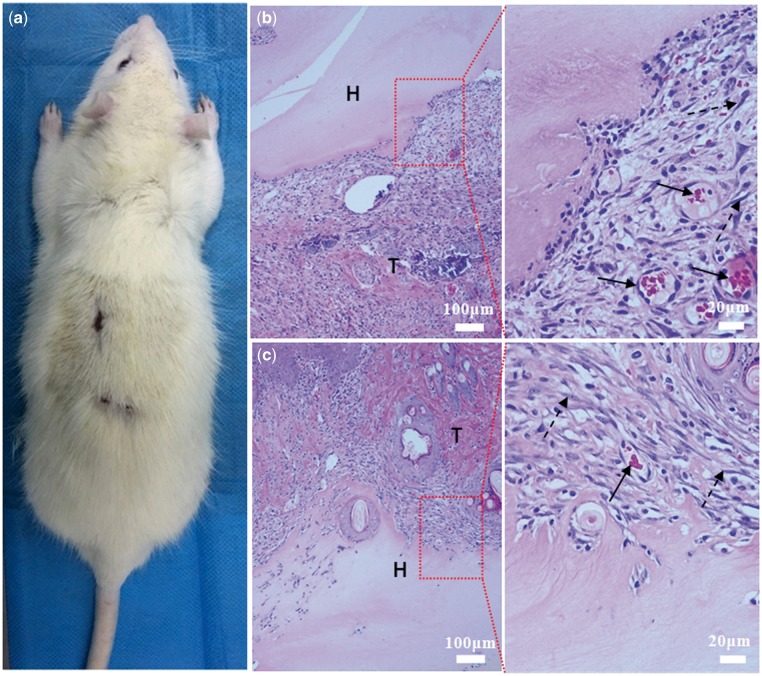
Histological sections of subcutaneous implantation of collagen/KGM composite hydrogel (**b**) and pure collagen hydrogel (**c**) 7 days after the operation. (**a**) rat for experiments. The magnified images are selected from the junctional zone between the embedded materials and the around tissues. T, tissues; H, hydrogel material. Solid arrow: neoformative capillaries. Dotted arrow: fibrocytes

On the first postoperative day, the activities and food intake of the experimental rats were normal without adverse reactions such as infection and suppuration. One week after the operation, the wounds had healed and the furs had grown out again. When the healed wounds were opened, it was observed that the implanted hydrogels had been degraded somewhat and the tissues around the implants had normal morphologies. The micrographs of HE section staining are shown in Fig. 11. The tissues around the implanted materials were of complete morphology, and no obvious inflammations occurred in the tissues implanted with the composite hydrogel or the pure collagen hydrogel, only a few inflammatory cells infiltrated in the junctional zone, indicating that the composite hydrogel of collagen/KGM maintains good biocompatibility equivalent to that of pure collagen. Additionally, in contrast with the tissues around the implant of the collagen hydrogel, a comparatively plenty of fibrocytes had proliferated in the tissues close to the composite hydrogel material, accompanied by the generation of a few neoformative capillaries, implying that the composite hydrogel of collagen/KGM has the potentials of promoting repairing and regeneration of wound tissues.

## Conclusion

In contrast to the blend films of collagen–KGM or collagen–KGM–polysaccharide obtained by solvent-casting method, a composite hydrogel of collagen/KGM has been successfully achieved under mild conditions based on the characteristic self-assembly behavior of collagen macromolecules. This preparation route of combining collagen with KGM in the form of hydrogel from the angle of collagen fibrillogenesis feature to obtain a composite hydrogel with collagen-based fibrillar networks is an original design for deeply exploring the composites of collagen and KGM. Compared with pure collagen hydrogel, the combination of KGM macromolecules into collagen matrix rendered such obtained composite hydrogel with enhanced mechanical properties and with a lower swelling ratio of the composite hydrogel-derived sponge scaffold. This composite hydrogel of collagen/KGM also exhibits biodegradability, excellent biocompatibility including cellular affinity and abilities of supporting normal cellular function expressing and promoting wound recovery. It is expected that the present study would provide a valuable approach to further develop the composite materials of collagen and KGM for biomedical applications. This developed composite hydrogels of collagen/KGM possessing shapeability and collagen bioactivities could find promising potentials as advanced supporting scaffolds for tissue engineering and medical reconstruction, and also as carrier and embedding materials for cells or drug delivery.

## Funding

The present study was supported by the National Natural Science Foundation of China (Grants 51373105 and 51203103), Sichuan Province Key Research and Development Project (2018SZ0046). 

*Conflict of interest statement*. None declared.
